# Local anaesthetic wound infiltration in addition to standard anaesthetic regimen in total hip and knee replacement: long-term cost-effectiveness analyses alongside the APEX randomised controlled trials

**DOI:** 10.1186/s12916-015-0389-1

**Published:** 2015-06-26

**Authors:** Elsa MR Marques, Ashley W. Blom, Erik Lenguerrand, Vikki Wylde, Sian M. Noble

**Affiliations:** School of Social and Community Medicine, University of Bristol, Canynge Hall, 39 Whatley Road, Bristol, BS8 2PS UK; Musculoskeletal Research Unit, School of Clinical Sciences, University of Bristol, Learning and Research Building, Southmead Hospital, Bristol, BS10 5NB UK

**Keywords:** Cost-effectiveness, Cost-utility, Local anaesthetic wound infiltration, Total hip replacement, Total knee replacement, Trial-based economic evaluation

## Abstract

**Background:**

The Arthroplasty Pain Experience (APEX) studies are two randomised controlled trials in primary total hip (THR) and total knee replacement (TKR) at a large UK orthopaedics centre. APEX investigated the effect of local anaesthetic wound infiltration (LAI), administered before wound closure, in addition to standard analgesia, on pain severity at 12 months. This article reports results of the within-trial economic evaluations.

**Methods:**

Cost-effectiveness was assessed from the health and social care payer perspective in relation to quality adjusted life years (QALYs) and the primary clinical outcome, the WOMAC Pain score at 12-months follow-up. Resource use was collected from hospital records and patient-completed postal questionnaires, and valued using unit cost estimates from local NHS Trust finance department and national tariffs. Missing data were addressed using multiple imputation chained equations. Costs and outcomes were compared per trial arm and plotted in cost-effectiveness planes. If no arm was dominant (i.e., more effective and less expensive than the other), incremental cost-effectiveness ratios were estimated. The economic results were bootstrapped incremental net monetary benefit statistics (INMB) and cost-effectiveness acceptability curves. One-way deterministic sensitivity analyses explored any methodological uncertainty.

**Results:**

In both the THR and TKR trials, LAI was the dominant treatment: cost-saving and more effective than standard care, in relation to QALYs and WOMAC Pain. Using the £20,000 per QALY threshold, in THR, the INMB was £1,125 (95 % BCI, £183 to £2,067) and the probability of being cost-effective was over 98 %. In TKR, the INMB was £264 (95 % BCI, −£710 to £1,238), but there was only 62 % probability of being cost-effective. When considering an NHS perspective only, LAI was no longer dominant in THR, but still highly cost-effective, with an INMB of £961 (95 % BCI, £50 to £1,873).

**Conclusions:**

Administering LAI is a cost-effective treatment option in THR and TKR surgeries. The evidence, because of larger QALY gain, is stronger for THR. In TKR, there is more uncertainty around the economic result, and smaller QALY gains. Results, however, point to LAI being cheaper than standard analgesia, which includes a femoral nerve block.

**Trial registration:**

ISRCTN96095682, 29/04/2010.

**Electronic supplementary material:**

The online version of this article (doi:10.1186/s12916-015-0389-1) contains supplementary material, which is available to authorized users.

## Background

Total hip (THR) and knee replacements (TKR) are common elective procedures with over 150,000 performed annually in the UK NHS [1]. In the USA in 2010, the estimated numbers of hospital discharges after THR and TKR procedures were 332,000 and 719,000, respectively [2]. For most patients with advanced osteoarthritis, THR and TKR are effective in treating pain and restoring physical function. However, some patients report chronic joint pain after surgery. Evidence suggests that around 20 % of patients with TKR and 10 % of patients with THR report an unfavourable pain outcome at between 3 months and 5 years post-operative [3].

Local anaesthetic wound infiltration (LAI), administered intra-operatively before wound closure, can provide better short-term pain relief and decrease hospital length of stay, but long term effectiveness and cost-effectiveness evidence is lacking [4]. In a recent systematic review of LAI, most studies reported outcomes up to 3 months post-surgery, and none of the studies included an economic evaluation of the intervention [4]. The Arthroplasty Pain Experience (APEX) studies are two randomised controlled trials, conducted at a large UK orthopaedic centre, which investigated the effect of intra-operative LAI, in addition to the standard analgesia, on pain severity at 12 months following primary THR and TKR for osteoarthritis [5].

The aim of this article is to report the results of the two within-trial economic evaluations. We assess the cost-effectiveness of LAI, in addition to the usual analgesia regimen, over the 12 months following surgery, from a health and social care payer perspective. In secondary analyses, we explore the economic results using only a health care payer perspective. In sensitivity analyses, we explore uncertainty around costing assumptions. This article follows the CHEERS reporting guidelines (Additional file 1: Appendix 1) [6].

## Methods

The APEX trials were approved by Southampton and South West Hampshire Research Ethics Committee (09/H0504/94) and all participants provided written informed consent. The trials were registered as an International Standardised Randomized Controlled Trial (96095682) and as a Clinical Trial of an Investigational Medicinal Product with the Medicine Healthcare and Regulatory Authority (18524/0215/001-0001) and EudraCT (2009– 013817–93).

The economic evaluations took a health and social care payer perspective: the NHS and Personal Social Services (PSS), in line with the National Institute for Health and Care Excellence (NICE) recommendations [7]. The APEX trials were two double-blinded single-centre trials in patients undergoing primary THR or TKR for osteoarthritis [8]. Patients were randomised to receive intra-operative LAI, administered before wound closure, in addition to the standard anaesthetic regimen, or standard anaesthesia alone. Standard anaesthetic care consisted of a spinal anaesthetic alone or in combination with sedation/light general anaesthetic for patients undergoing THR surgery. In TKR, standard care also included administering a femoral nerve block in addition to spinal or general anaesthesia. The primary clinical outcome was the Western Ontario and McMaster Universities Osteoarthritis Index (WOMAC) Pain Scale at 12 months post-operative [9].

The primary economic analyses alongside the APEX trials were cost-utility analyses, whereby incremental costs were compared with incremental quality adjusted life years (QALYs) gained, at 12 months after surgery. The secondary economic analyses compared the incremental costs with increment health benefits measured by the WOMAC Pain score at 12 months follow-up. In addition, we presented results in relation to both health outcomes from an NHS perspective only.

### Health outcomes

The primary health outcome for the APEX trials economic evaluation was the QALY. A QALY is a measure of disease burden that weights survival by quality of life. This generic measure allows for direct effectiveness and cost-effectiveness comparisons between interventions across all patient groups and health conditions. NICE guidelines provide recommendations for UK’s societal willingness to pay for one QALY gained [10], which allows for inferences about cost-effectiveness of interventions to be made. QALYs for the APEX trials were derived using the EuroQoL questionnaire (EQ-5D-3L) [11] completed by patients at baseline, and at 3, 6, and 12 months follow-up. The EQ-5D-3L questionnaire comprises five dimensions: mobility, self-care, usual activities, pain/discomfort, and anxiety/depression. Each dimension has three levels: no problems, some problems, or severe problems.

The primary clinical outcome was the WOMAC Pain score, completed by patients at 12 months after surgery. WOMAC Pain scores ranged from 0–100, with lower scores indicating more severe pain.

### Resource use identification and collection

Collection of resource use data was identical for both the THR and TKR trials and related to services used for reasons related to the patients’ joint replacement. Resources used at the treating hospital were extracted from medical records onto study-specific proformas. These included the initial inpatient stay for joint replacement, and subsequent inpatient stays and outpatient visits during the 12 months of follow-up. Initial inpatient resource use included operating theatre time, intra-operative LAI injection in the intervention group, time spent in recovery, and number of days admitted to a ward after surgery. After initial hospital discharge, inpatient and outpatient resource use data collected included the duration, reason for visit and ward details of inpatient, day-case admissions, accident and emergency visits, and outpatient visits at clinics.

All other resource use was collected using patient-completed questionnaires, administered by post at 3, 6, and 12 months follow-up. This included secondary care at other hospitals, community-based health care visits, medications use, and use of social services. Community health care included contacts with a doctor, practice nurse, district nurse, community physiotherapist, and occupational therapist contacts. Social services included food at home and home care worker services, contacts with social workers, equipment provided to patients, and changes made to patients’ homes during the follow-up period. We excluded services, equipment, and home changes paid for privately by patients.

Patients were provided with resource use logs at hospital discharge, and at 3 and 6 months to facilitate their completion of these questionnaires [12]. Both the logs and the questionnaires were tailored to the type of joint replacement (THR or TKR). Examples of the 3-months resource use questionnaire and log are available online on the DIRUM (Data Instruments for Resource Use Measurement) database [13, 14].

### Valuation of resource use

Resources used during the initial hospital stay were valued using unit costs obtained from the North Bristol Trust finance department. Cost estimates for time spent in theatre, recovery, and admissions to hospital wards included staff time, overheads, consumables, and medications. Unit costs for the LAI injection were provided by the Management and Procurement Department at North Bristol NHS Trust.

For secondary care visits in the 12 months follow-up period, we used information on the reason for inpatient admissions, duration of episode and clinical expert advice to derive healthcare resource group codes. Healthcare resource groups and outpatient appointments by clinical specialty, were valued using Department of Health Reference costs [15].

Community-based resources and personal social services were valued using Curtis’ unit costs for health and social care [16]. Equipment and changes to patients’ homes, such as dressing aids, furniture raisers, walking aids, and chair lifts, were financed by social services, but provided to patients, on loan, through occupational therapists and physiotherapists at North Bristol Trust. We assumed the useful life of the equipment to be 2 years and valued it as the fraction of equipment cost proportional to the duration of patient use. Unit costs were obtained from equipment suppliers to North Bristol Trust or online sources from other suppliers when procurement costs were not available. Prescribed medications were valued using the British National Formulary 68 [17]. Table 1 describes sources of unit cost estimates.

**Table 1:** Unit costs for total hip and knee replacement resource use

Resource use	Unit cost	Assumption	Source
Initial inpatient admission			
Theatre (per minute)	£14.22	Includes implant cost, staff time, overheads, consumables, facilities	North Bristol NHS Trust Finance department
Injection of local anaesthesia infiltration (LAI)	£2.00	Box of bupivicaine with adrenaline 0.25 %/1 in 200,000 is £20.00; one box contains 10 ampoules	North Bristol NHS Trust Finance department, management, and procurement
Recovery (per minute)	£3.84	Includes staff time with overheads, consumables, facilities, and medications administered during stay; base cost per minute	North Bristol NHS Trust Finance department
Day in general orthopaedics ward: Frome, Severn, Kennett, and Cardiac Care Unit	£311	Includes staff time with overheads, consumables, facilities, and medications administered during stay; base cost per day	North Bristol NHS Trust Finance department
Day in other orthopaedics ward: Chew	£250	Includes staff time with overheads, consumables, facilities, and medications administered during stay; base cost per day	North Bristol NHS Trust Finance department
Day in high dependency unit	£1,356	Includes staff time with overheads, consumables, facilities, and medications administered during stay; base cost per day	North Bristol NHS Trust Finance department
Inpatient admissions following discharge from initial surgery			
Revision surgery – Total knee replacement (TKR)	£9,439	HB22A major knee procedures for non-trauma, with major comorbidity and complication (CC)	NHS Reference Costs 2012–2013: Non-elective long stay
Revision surgery – Total hip replacement (THR)	£8,890	HB12A major hip procedures for non-trauma, with major CC	NHS Reference Costs 2012–2013: Non-elective long stay
Manipulation under anaesthetic – TKR	£2,044	HB24C minor knee procedures for non-trauma, category 2, without CC	NHS Reference Costs 2012–2013: Non-elective long stay
Infections	£4,124	Infections of bones or joints, with CC Score 5–8	NHS Reference Costs 2012–2013: Non-elective long stay
Day case procedures – TKR	£655	Day case: HB29Z minimal knee procedures for non-trauma	NHS Reference Costs 2012–2013: Non-elective day case
Day case procedures – THR	£788	Day case: HB19Z minimal hip procedures for non-trauma	NHS Reference Costs 2012–2013: Non-elective day case
Nights in hospital for other admissions ^a^	£311	Unit cost based on SMH cost per night in general orthopaedics ward	North Bristol NHS Trust Finance department
A and E and outpatient visits			
Accident and emergency	£117	Average of all accident and emergency visits	NHS Reference Costs 2012–2013: Outpatient appointments: 180 Accident & Emergency
Trauma and orthopaedics – consultant led	£102	Non-admitted face to face attendance, follow-up, consultant led	NHS Reference Costs 2012–2013: Outpatient appointments: 110 Trauma & Orthopaedics
Trauma and orthopaedics – non-consultant led	£90	Non-admitted face to face attendance, follow-up, non-consultant led	NHS Reference Costs 2012–2013: Outpatient appointments: 110 Trauma & Orthopaedics
Physiotherapy – non-consultant led	£39	Non-admitted face to face attendance, follow-up, non-consultant led	NHS Reference Costs 2012–2013: Outpatient appointments: 650 Physiotherapy
General Medicine – consultant led	£145	Non-admitted face to face attendance, follow-up, consultant led	NHS Reference Costs 2012–2013: Outpatient appointments: 300 General Medicine
Neurology – consultant led	£157	Non-admitted face to face attendance, follow-up, consultant led	NHS Reference Costs 2012–2013: Outpatient appointments: 400 Neurology
Respiratory – consultant led	£137	Non-admitted face to face attendance, first appointment, consultant led	NHS Reference Costs 2012–2013: Outpatient appointments: 340 Respiratory Medicine
Pain management – consultant led	£136	Non-admitted face to face attendance, follow-up, consultant led	NHS Reference Costs 2012–2013: Outpatient appointments: 191 Pain management
Vascular – consultant led	£133	Non-admitted face to face attendance, follow-up, consultant led	NHS Reference Costs 2012–2013: Outpatient appointments: 107 Vascular surgery
Dermatology – consultant led	£95	Non-admitted face to face attendance, follow-up, consultant led	NHS Reference Costs 2012–2013: Outpatient appointments: 330 Dermatology
Haematology – consultant led	£209	Non-admitted face to face attendance, follow-up, consultant led	NHS Reference Costs 2012–2013: Outpatient appointments: 303 Clinical Haematology
Community based health services			
GP surgery visit	£45	Base cost per patient contact with GP with qualifications, including direct care staff costs, lasting 11.7 min	Personal Social Services Research Unit (PSSRU) 2013: 10.8b General practitioner
GP home visit	£114	Base cost per out of surgery visit with GP with qualifications, including direct care staff costs, lasting 23.4 min	PSSRU 2013: 10.8b General practitioner
Phoned GP for advice	£27	Base cost per telephone consultation with GP with qualifications, including direct care staff costs, lasting 7.1 min	PSSRU 2013: 10.8b General practitioner
GP Practice nurse visit	£13.43	Based on 15.5 min per surgery consultation using the base cost (£52) of one hour of face-to-face contact with GP nurse with qualifications	PSSRU 2013: 10.6 Nurse (GP practice)
Phoned GP practice nurse for advice	£4	Based on 6 min of GP nurse time using the base cost (£40) of 1 hour of GP nurse time with qualifications	PSSRU 2013: 10.6 Nurse (GP practice)
Repeat prescription (without seeing doctor)	£11.40	Based on 3 min of GP time, using the base cost of 1 minute GP patient contact time (£3.80), with qualifications, including direct care staff costs	PSSRU 2013: 10.8b General practitioner
District nurse	£18.08	Based on the assumption that the duration of a district nurse visit is the same as GP nurse visit (15.5 min) and using the base cost of 1 hour of community nurse visit (£70) with qualifications including travel	PSSRU 2013: 10.1 Community nurse
Occupational therapist at home/GP surgery/clinic	£17	Based on 30 min contact using the base cost (£34) of 1 hour of occupational therapist contact with qualifications	PSSRU 2013: 9.2 NHS community occupational therapist
Community physiotherapist at home/GP surgery/clinic	£17	Based on 30 min contact using the base cost (£34) of 1 hour of physiotherapist contact with qualifications	PSSRU 2013: 9.1 Community physiotherapist
Prescriptions costs per consultation	£44.64	Prescription costs per consultation (net ingredient cost)	PSSRU 2013:10.8b General practitioner
Social services			
Home care worker (home help) provided by social services	£24	Based on 1 hour of face-to-face weekday contact for independent sector home care provided for social services	PSSRU 2013: 11.6
Food at home service (meals on wheels)	£3.14	Based on one meal a day using the Meals on Wheels average weekly cost (2012/2013) of £44, assuming two meals per day, 7 days a week	PSSRU 2013: 8.1.1 Community care package for older people: very low cost
Social worker visits	£113	Based on a 30 min visit using the base cost (£226) of 1 hour of face-to-face contact of social worker with qualifications	PSSRU 2013: 11.2 Social worker (adult services)
Social worker telephone calls	£39.50	Based on a 30 min telephone call using a base cost (£79) of 1 hour of client-related work of a social worker with qualifications	PSSRU 2013: 11.2 Social worker (adult services)
Home changes and equipment provided by social services:		All unit costs for home changes and equipment are based on 3-month loan period, assuming a 24 months life span	
Toilet seat or toilet raiser	£1.80	Cost of equipment £14	NRS price – equipment provider for North Bristol NHS Trust
Dressing aids: socks, shoes, etc.	£1.25	Cost of equipment £10	NRS price – equipment provider for North Bristol NHS Trust
Furniture raisers	£2.48	Cost of equipment £20	NRS price – equipment provider for North Bristol NHS Trust
Perching stool	£6.00	Cost of equipment £48	NRS price – equipment provider for North Bristol NHS Trust
Walker or trolley	£7.50	Cost of equipment £60	NRS price – equipment provider for North Bristol NHS Trust
Crutches	£3.75	Cost of equipment £30	NRS price – equipment provider for North Bristol NHS Trust
Commode	£5.69	Cost of equipment £46	NRS price – equipment provider for North Bristol NHS Trust
Rails and hand grips	£2.85	Cost of equipment £23	NRS price – equipment provider for North Bristol NHS Trust
Bath boards	£3.00	Cost of equipment £24	NRS price – equipment provider for North Bristol NHS Trust
Hospital bed at home	£59.88	Cost of equipment £479	Online search for procurement prices (cheaper range)
Bath lift	£44.75	Cost of equipment £358	Online search for procurement prices (cheaper range)
Chair and stair lift	£125.00	Cost of equipment £1000	Online search for procurement prices (cheaper range)

^a^ When healthcare resource group codes could not be derived because of insufficient information, inpatient admissions were valued multiplying the number of nights admitted to a ward by the cost of a night in a general orthopaedics ward provided by the North Bristol Trust finance department

### Data analysis

The two separate economic evaluations, for THR and TKR, were intention-to-treat analyses and used the same methodology. QALYs were derived for each treatment group, attributing the quality weights from a sample of the UK population to the patients answers to the EQ-5D-3L questionnaire [18], at baseline (pre-operative), and at 3, 6, and 12 months follow-up. QALYs were then estimated using the “area under the curve approach”, which assumes a linear change between time points [19]. Mean and standard deviations for QALYs were calculated for each group.

Costs were estimated by multiplying units of resource use by their unit cost, reported in 2012–2013 prices (Table 1). Resources were categorised into 17 categories. The total cost for each patient for each of these categories was calculated as the sum of the cost of the resource use items. For each category using all available data, we calculated means and standard deviations for resource use and costs by treatment group. The cost categories were then grouped into initial inpatient stay costs, secondary care costs during the follow-up period, community-based health care costs including medication, and personal social service (PSS) costs. The total individual patient cost for these four groups, as well as total NHS costs and total NHS and PSS costs, were calculated as above. Costs and outcomes were not discounted because of the 1 year duration of follow-up.

Incremental costs for the four main cost groupings, and QALY and WOMAC differences between groups, were then estimated using ordinary least squares regression, adjusting for APEX trial treatment group allocation and randomisation minimisation variables: baseline WOMAC Pain score and surgical approach. QALYs were further adjusted for baseline utility imbalances [19].

Missing cost and outcome data were imputed using chained equations for multiple imputation [20], and Royston’s ‘ice’ command in Stata v13 [21], to generate 20 complete datasets. This method uses regression techniques to estimate missing values, based on the values of available data. Complete datasets for the primary clinical outcome were taken from the statistical analysis of the trial outcomes [5]. The 17 cost categories and four EQ-5D utility scores (baseline and three follow-up time points) were imputed jointly, by treatment group allocation, adjusting for the primary clinical outcome, trial minimisation variables, and patient baseline characteristics: age, sex, body mass index, and dichotomous variables for education level (high vs medium or low) and marital status (single vs married or other). QALYs and grouped cost categories were then recalculated using the imputed values, and incremental costs and both outcomes with imputed data were then re-estimated adjusting for the same variables as described in the regression models above.

The incremental costs and outcomes (QALYs and WOMAC Pain scores) were presented in a cost-consequences table and depicted on cost-effectiveness planes. If no treatment was dominant, an incremental cost-effectiveness ratio (ICER) was estimated [22]. The ICER is a ratio that divides the difference in mean costs between arms by the difference in mean outcomes.

The incremental net monetary benefits (INMB) of the intervention were estimated in relation to QALYs, given that this health outcome has recommended UK societal willingness-to-pay thresholds [10]. The INMB statistic is estimated by multiplying the incremental health gain observed in the intervention, compared with control, by the societal willingness-to-pay thresholds for that health gain (λ), and then deducting the incremental cost difference [23]. We used thresholds of £10,000 per QALY, a threshold closer to recent valuations of QALYs for the UK [24], and the NICE recommendations of £20,000 and £30,000 per QALY [7]. Positive INMB statistics indicate a cost-effective intervention, whereby society is willing to pay more for the health gain than what the intervention costs. To account for the uncertainty around the economic results, bootstrapped confidence intervals (BCI) with 1,000 replications were estimated for the adjusted costs, outcomes, the INMB statistics, and ICERs. Bootstrapped costs and effects were depicted in cost-effectiveness planes [25]. In cost-effectiveness acceptability curves (CEACs), we illustrated the probability of the intervention being cost-effective, given a range of societal willingness-to-pay thresholds. All analyses were conducted using Stata v13 [26]. In secondary analysis, we presented the results from a narrower perspective: the NHS perspective only.

### Sensitivity analyses

In one-way deterministic sensitivity analyses, we explored methodological and sample uncertainty around the economic results in relation to QALYs and the WOMAC Pain score. In the first scenario, we assumed a macro-costing approach to the prescribed medications, using the national average value of prescriptions costs per general practitioner (GP) consultation [16], and multiplying it by the number of the GP consultations attended by the patient. Secondly, we explored the potential variation in the local trust cost estimates for the initial inpatient stay: theatre and recovery costs, LAI injection, and daily admission rates to wards, using worst and best case scenarios where local costs could be up to 50 % higher, or 50 % lower, than our local trust. In the THR trial, we explored the variation in the results from excluding two high cost patients: one patient in the control group, who was an intensive user of home care help, and one patient in the intervention group, who required further surgeries with high hospital re-admission costs. All sensitivity analyses were performed from NHS and PSS perspectives. Imputation models for all cost categories and utility scores were redone accounting for changes in sensitivity analysis.

## Results and discussion

### The APEX trials

The APEX trials recruited 322 patients undergoing THR and 316 patients undergoing TKR between November 2009 and February 2012. In the THR trial, patients in the intervention group had less pain at 12 months post-operatively, and were more likely to report none to moderate pain than severe pain compared to the standard care group. In the TKR trial, there was no strong evidence that LAI influenced pain severity at 12 months post-operative. The clinical outcomes of the APEX trials are reported in full elsewhere [5].

In the THR trial, 88/163 (54 %) patients in the intervention group and 85/159 (53 %) patients in the standard care group had complete NHS and PSS cost data (Table 2). In the TKR trial, the corresponding figures were 70/157 (45 %) for the intervention group, and 75/159 (47 %) for the control group (Table 3). Complete cost and QALY data were available for 159 patients in the THR trial (49 %) and 142 patients in the TKR trial (45 %; not reported). Given the amount of missing data, our primary economic results statistics included imputed missing cost and outcome data.

**Table 2:** Mean resource use and cost by APEX trial treatment group for total hip replacements (available cases)

	Intervention					Control				
	N	Mean resource use	(SD)	Mean cost	(SD)	N	Mean resource use	(SD)	Mean cost	(SD)
Initial inpatient stay										
Theatre time (in minutes)	148	99	29	£1,407	£411	147	101	31.6	£1,441	£449
Recovery time (in minutes)	143	103	65	£397	£251	144	113	77.4	£435	£297
Days in wards	153	5.2	3.3	£1,597	£1,516	154	5.2	2.8	£1,553	£886
Secondary care after initial discharge										
Inpatient admissions after initial discharge ^a^	115			£341	£1,847	122			£101	£554
Orthopaedics appointments	142	1.96	1.2	£199	£121	146	1.97	1.4	£201	£138
Physiotherapy appointments	142	0.19	0.8	£7	£32	146	0.23	0.8	£9	£30
Accident and emergency visits	142	0.06	0.4	£7	£46	146	0.04	0.3	£5	£30
Other appointments	142	0.04	0.3	£5	£37	146	0.04	0.3	£7	£59
Community-based resources										
GP contacts combined	107	1.90	3.3	£61	£113	108	2.66	4.5	£83	£145
Nurse contacts combined	110	1.60	4.1	£25	£70	114	1.24	2.7	£18	£41
Occupational therapist contacts combined	113	0.04	0.4	£1	£7	116	0.08	0.5	£1	£8
Community physiotherapist contacts combined	109	0.25	1.1	£4	£19	113	0.58	1.8	£10	£30
Prescribed medications ^a^	113			£22	£77	120			£21	£55
Total NHS cost ^b^	92			£3,773	£1,557	87			£3,762	£1,065
Personal social services (PSS)										
Home care worker (in hours)	139	1.11	8.1	£27	£195	144	5.36	56.3	£129	£1,351
Meals (food at home services)	137	2.76	24.0	£9	£75	138	0.00	0.0	£0	£0
Contacts with social worker	138	0.05	0.5	£4	£36	144	0.13	1.1	£7	£59
Home changes ^a^	161			£1	£3	158			£2	£5
Total NHS+PSS cost ^b^	88			£3,837	£1,642	85			£3,948	£2,108

^a^ The category combines different types of resource use, therefore an overall mean resource use could not be calculated

^b^ Total costs computed for patients with complete cost categories

**Table 3:** Mean resource use and cost by APEX trial treatment group for total knee replacements (available cases)

	Intervention					Control				
	N	Mean resource use	(SD)	Mean cost	(SD)	N	Mean resource use	(SD)	Mean cost	(SD)
Initial inpatient stay										
Theatre time (in minutes)	142	102	32	£1,449	£453	145	103	32.9	£1,461	£469
Recovery time (in minutes)	140	94	44	£359	£169	136	104	69.1	£398	£265
Days in wards	147	5.9	3.9	£1,789	£1,224	149	5.2	2.9	£1,586	£1,034
Secondary care after initial discharge										
Inpatient admissions after initial discharge ^a^	103			£104	£533	110			£296	£907
Orthopaedics appointments	128	2.06	1.5	£209	£149	137	1.99	1.4	£202	£143
Physiotherapy appointments	128	0.44	2.1	£17	£82	137	0.40	1.3	£16	£50
Accident and emergency visits	128	0.16	0.7	£18	£84	137	0.18	1.2	£20	£145
Other appointments	128	0.00	0.0	£0	£0	137	0.03	0.2	£4	£34
Community Based resources										
GP contacts combined	85	2.65	4.3	£84	£151	102	3.83	5.7	£122	£212
Nurse contacts combined	90	0.98	1.4	£14	£22	104	1.09	2.7	£16	£43
Occupational therapist contacts combined	95	0.28	1.1	£5	£19	105	0.25	1.3	£4	£22
Community physiotherapist contacts combined	90	1.03	2.7	£18	£45	107	1.29	3.5	£22	£60
Prescribed medications ^a^	102			£61	£211	108			£48	£81
Total NHS cost ^b^	70			£3,807	£1,277	78			£4,255	£1,804
Personal social services (PSS)										
Home care worker (in hours)	135	1.17	12.9	£28	£310	136	1.24	14.4	£30	£346
Meals (food at home services)	132	0.14	1.6	£0	£5	129	0.11	1.2	£0	£4
Contacts with social worker	133	0.11	1.1	£4	£45	134	0.14	1.6	£7	£84
Home changes ^a^	157			£3	£13	158			£1	£4
Total NHS+PSS cost ^b^	70			£3,811	£1,276	75			£4,303	£2,102

^a^ The category combines different types of resource use, therefore an overall mean resource use could not be calculated

^b^ Total costs computed for patients with complete cost categories

### Resource use and costs

Tables 2 and 3 show the observed mean and standard deviations for the resource use and costs by treatment group, and by resource use category, for all participants with data. All available data is reported for each category.

For both the THR and TKR trials, the available case results indicate that initial inpatient stay cost categories are similar between groups. Administering intra-operative LAI does not appear to increase operation time, but may reduce time in recovery by about 10 min. The length of stay following recovery was, on average, 5.2 days for both arms of the THR trial, and 5.9 days for the intervention group of the TKR trial compared to 5.2 days in the standard care group.

After initial discharge, there were lower readmission costs for TKR patients in the intervention group, whereas the reverse was true for patients receiving THR. Participants in both arms of the two trials had a similar number of appointments in the period.

In both trials, the intervention group seemed to have less contacts with a doctor compared with the control group, but more nurse contacts in the THR trial. Personal social services costs contributed a minor amount to the overall costs of delivering treatment for both types of joint replacement. Total unadjusted mean NHS and PSS cost was lower in the intervention group than the control group for both trials. All cost drivers for these trials display high variability, with large standard deviations around the categorical mean cost estimates.

### Adjusted outcomes and costs

Tables 4 (THR) and 5 (TKR) report the costs and outcome differences between arms. In THR, patients in the intervention arm had an incremental QALY gain of 0.052 (95 % BCI, 0.01 to 0.09) compared with the control group. This corresponded to patients in the intervention arm spending on average an estimated 19 more days in “perfect health” than patients in the control arm. In TKR, the estimated health benefit gain for the intervention arm was lower and findings were more uncertain, with a mean of 0.009 QALYs gained per patient in the intervention arm (95 % BCI, −0.04 to 0.057). In relation to the primary clinical result, patients in the intervention arm receiving THR experienced a greater reduction in pain severity at 12 months, by 5.35 points on the WOMAC Pain scale (95 % CI, 1.33 to 9.34) compared to the control arm. In TKR, there was weaker evidence for the pain reduction observed, with patients in the intervention arm experiencing less pain at 12 months by 3.33 points on the WOMAC Pain scale (95 % CI, −1.21 to 7.88).

**Table 4:** Total hip replacement: differences in costs and outcomes between APEX randomised groups

	Difference (intervention – control)		
	N	Mean	(95 % CI)
OUTCOMES			
QALYs			
QALY gain – available cases ^a^	216	0.064	(0.018 to 0.110)
QALY gain – imputed data ^b^	322	0.052	(0.011 to 0.094)
Primary clinical outcome			
WOMAC pain score improvement – available cases ^c^	281	4.74	(0.95 to 8.54)
WOMAC pain score improvement – imputed data ^d^	322	5.35	(1.33 to 9.34)
COST			
Initial inpatient stay			
Total of inpatient stay – available cases ^a^	273	-£123	(£–364 to £118)
Total of inpatient stay – imputed data ^b^	322	-£32	(£–349 to £285)
Secondary care after initial discharge			
Inpatient admissions – available cases ^a^	236	£251	(£–114 to £617)
Inpatient admissions – imputed data ^b^	322	£139	(£–174 to £452)
Outpatient visits – available cases ^a^	287	-£2	(£–36 to £32)
Outpatient visits with – imputed data ^b^	322	£4	(£–33 to £42)
Total secondary care cost – available cases ^a^	231	£251	(£–136 to £639)
Total secondary care cost – imputed data ^b^	322	£143	(£–184 to £471)
Community-based resources			
Total community-based costs – available cases ^a^	202	-£4	(£–51 to £43)
Total community-based costs – imputed data ^b^	322	-£34	(£–83 to £16)
Total NHS cost – complete NHS cost cases ^a^	179	£15	(£–373 to £404)
Total NHS cost – imputed data ^b^	322	£78	(£–404 to £560)
Personal social services (PSS)			
Total personal social services – available cases ^a^	263	-£83	(£–289 to £123)
Total personal social services – imputed data ^b^	322	-£164	(£–418 to £91)
Total NHS + PSS cost – complete NHS+PSS cost cases ^a^	173	-£94	(£–634 to £446)
Total NHS + PSS cost – imputed data ^b^	322	-£86	(£–634 to £462)

^a^ Adjusted for minimisation variables (baseline WOMAC pain score and surgical approach) and baseline utility for QALYs, robust standard errors

^b^ Adjusted for minimisation variables (and baseline utility for QALYs), robust standard errors, M=20 multiple imputation sets, bootstrapped confidence intervals with 1000 replications

^c^ Adjusted for minimisation variables (baseline WOMAC pain score and surgical approach) and baseline utility for QALYs

^d^ Adjusted for minimisation variables, M=20 multiple imputation sets from main statistical analysis

**Table 5:** Total knee replacement: differences in costs and outcomes between APEX randomised groups

	Difference (intervention – control)		
	N	Mean	(95 % CI)
OUTCOMES			
QALYs			
QALY gain – available cases ^a^	201	0.010	(−0.039 to 0.060)
QALY gain – imputed data ^b^	316	0.009	(−0.040 to 0.057)
Primary clinical outcome			
WOMAC pain score improvement – available cases ^c^	273	3.83	(−0.83 to 8.49)
WOMAC pain score improvement – imputed data ^d^	316	3.33	(−1.21 to 7.88)
COST			
Initial inpatient stay			
Total of inpatient stay – available cases ^a^	268	£89	(£–194 to £371)
Total of inpatient stay – imputed data ^b^	316	£152	(£–140 to £444)
Secondary care after initial discharge			
Inpatient admissions – available cases ^a^	213	-£170	(£–365 to £24)
Inpatient admissions – imputed data ^b^	316	-£239	(£–489 to £11)
Outpatient visits – available cases ^a^	265	£2	(£–47 to £52)
Outpatient visits with – imputed data ^b^	316	£13	(£–43 to £70)
Total secondary care cost – available cases ^a^	203	-£165	(£–391 to £61)
Total secondary care cost – imputed data ^b^	316	-£226	(£–485 to £34)
Community-based resources			
Total community based costs – available cases ^a^	170	-£56	(£–142 to £31)
Total community based costs – imputed data ^b^	316	£0	(£–99 to £99)
Total NHS cost – complete NHS cost cases ^a^	148	-£343	(£–822 to £137)
Total NHS cost – imputed data ^b^	316	-£74	(£–490 to £343)
Personal social services (PSS)			
Total personal social services – available cases ^a^	259	-£4	(£–95 to £87)
Total personal social services – imputed data ^b^	316	-£4	(£–89 to £82)
Total NHS + PSS cost – complete NHS+PSS cost cases ^a^	145	-£404	(£–924 to £117)
Total NHS + PSS cost – imputed data ^b^	316	-£77	(£–528 to £374)

^a^ Adjusted for minimisation variables (baseline WOMAC c pain score and surgical approach) and baseline utility for QALYs, robust standard errors

^b^ Adjusted for minimisation variables (and baseline utility for QALYs), robust standard errors, M=20 multiple imputation sets, bootstrapped confidence intervals with 1000 replications

^c^ Adjusted for minimisation variables (baseline WOMAC pain score and surgical approach) and baseline utility for QALYs

^d^ Adjusted for minimisation variables, M=20 multiple imputation sets from main statistical analysis

In both THR and TKR, differences in the imputed and adjusted NHS, and NHS and PSS costs, indicated that patients in the intervention group had lower mean costs than those in the standard care group at 1 year. In THR, the mean cost per patient in the intervention group was £32 lower for the initial inpatient stay (95 % BCI, £–349 to £285), £34 lower for community-based health care costs (95 % BCI, £–83 to £16), and £139 more for readmission costs (95 % BCI, £–174 to £452), when compared with the control group, with mean overall NHS costs higher by £78 (95 % BCI, £–404 to £560). Mean PSS costs were lower in the intervention arm by £164 per patient (95 % BCI, £–418 to £91). Therefore, the combined NHS and PSS mean cost per patient was £86 lower in the intervention group (95 % BCI, £–634 to £462).

In contrast, in TKR, the mean cost per patient for the initial inpatient stay in the intervention arm was greater by £152 (95 % BCI, £–140 to £444) and the mean cost per patient of hospital readmissions was lower by £239 (95 % BCI, £–489 to £11). Therefore, there was an overall lower combined NHS and PSS mean cost of £77 (95 % BCI, £–528 to £374) per patient in the intervention group compared with the control group.

### Economic results: NHS and PSS perspective

The cost and outcome results indicate that LAI, in addition to standard analgesia, is the dominant treatment option in the two trials: cost-saving and more effective, both in relation to QALYs and pain severity at 12 months, than current clinical practice. Tables 6 and 7 present the cost-effectiveness results in THR and TKR, respectively. Given that the intervention was dominant, no incremental cost-effectiveness ratios were calculated for the base case results.

**Table 6:** Total hip replacement economic results

	Difference (intervention – control) ^a^			
	N	Mean	(95 % CI)	*P* value
MAIN ANALYSIS: NHS and personal social services (PSS) perspective				
Mean QALY gain	322	0.052	(0.017 to 0.087)	0.004
Mean NHS+PSS cost difference	322	–£86	(£–571 to £399)	0.730
Incremental net monetary benefit – lambda £10,000	322	£606	(£–55 to £1,266)	0.072
Incremental net monetary benefit – lambda £20,000	322	£1,125	(£183 to £2,067)	0.019
Incremental net monetary benefit – lambda £30,000	322	£1,645	(£385 to £2,905)	0.011
SECONDARY ANALYSIS: NHS perspective only				
Mean NHS cost difference	322	£78	(£–347 to £502)	0.720
Incremental net monetary benefit – lambda £20,000	322	£961	(£50 to £1,873)	0.039
Incremental Cost per point in WOMAC pain decrease ^b^	322	£16	(£–16,591 to £16,622)^c^	0.999
SENSITIVITY ANALYSIS: Using macro-costed prescribed medications				
Prescribed medications ^b^	322	–£24	(£–28 to £–20)	
Mean QALY gain	322	0.052	(0.017 to 0.087)	0.004
Mean NHS+PSS cost difference	322	–£107	(£–590 to £376)	0.660
Incremental Net Monetary Benefit – lambda £20,000	322	£1,666	(£406 to £2,925)	0.010
SENSITIVITY ANALYSIS: 50 % higher local inpatient costs				
Initial inpatient stay ^b^	322	–£50	(£–156 to £56)	
Mean QALY gain	322	0.052	(0.017 to 0.088)	0.004
Mean NHS+PSS cost difference	322	–£106	(£–697 to £485)	0.730
Incremental net monetary benefit – lambda £20,000	322	£1,151	(£121 to £2,181)	0.028
SENSITIVITY ANALYSIS: 50 % lower local inpatient costs				
Initial inpatient stay ^b^	322	–£8	(£–44 to £27)	
Mean QALY gain	322	0.050	(0.015 to 0.086)	0.006
Mean NHS+PSS cost difference	322	–£44	(£–445 to £358)	0.830
Incremental net monetary benefit – lambda £20,000	322	£1,051	(£164 to £1,938)	0.020
SENSITIVITY ANALYSIS: dropping high cost patients				
Mean QALY gain	320	0.052	(0.016 to 0.088)	0.004
Mean NHS+PSS cost difference	320	–£73	(£–449 to £302)	0.70
Incremental net monetary benefit – lambda £20,000	320	£1,121	(£ 215 to £2,026)	0.015

^a^ All results are adjusted for minimisation variables, and baseline utility for QALYs, M=20 multiple imputation sets, bootstrapped confidence intervals with 1000 replications, except results noted with ^b^

^b^ Unadjusted estimates with imputed data using M=20 multiple imputation sets

^c^ This confidence interval includes negative ICER values. These negative values indicate that the intervention is dominant

**Table 7:** Total knee replacement economic results

	Difference (intervention – control) ^a^			
	N	Mean	(95 % CI)	*P* value
MAIN ANALYSIS: NHS and personal social services (PSS) perspective				
Mean QALY gain	316	0.009	(−0.030 to 0.049)	0.64
Mean NHS+PSS Cost difference	316	–£77	(£–451 to £296)	0.68
Incremental net monetary benefit – lambda £10,000	316	£171	(£–452 to £793)	0.59
Incremental net monetary benefit – lambda £20,000	316	£264	(£–710 to £1,238)	0.60
Incremental net monetary benefit – lambda £30,000	316	£357	(£–992 to £1,707)	0.60
SECONDARY ANALYSIS: NHS perspective only				
Mean QALY gain	316	0.009	(−0.030 to 0.049)	0.64
Mean NHS cost difference	316	–£74	(£–414 to £266)	0.67
Incremental net monetary benefit – lambda £20,000	316	260	(£–690 to £1,210)	0.59
SENSITIVITY ANALYSIS: using macro-costed prescribed medications				
Prescribed medications ^b^	316	–£14	(£–21 to £ –6)	
Mean QALY gain	316	0.009	(−0.030 to 0.049)	0.64
Mean NHS+PSS cost difference	316	–£121	(£–491 to £249)	0.52
Incremental net monetary benefit – lambda £20,000	316	£308	(£–665 to £1,281)	0.53
SENSITIVITY ANALYSIS: 50 % higher local inpatient costs				
Initial inpatient stay ^b^	316	–£258	(£164 to £353)	
Mean QALY gain	316	0.008	(−0.032 to 0.047)	0.70
Mean NHS+PSS cost difference	316	–£2	(£–483 to £478)	0.99
Incremental net monetary benefit – lambda £20,000	316	£159	(£–882 to £1,200)	0.76
SENSITIVITY ANALYSIS: 50 % lower local inpatient costs				
Initial inpatient stay ^b^	316	£84	(£–66 to £234)	
Mean QALY gain	316	0.006	(−0.034 to 0.045)	0.79
Mean NHS+PSS cost difference	316	–£143	(£–423 to £137)	0.32
Incremental net monetary benefit – lambda £20,000	316	£253	(£–674 to £1,180)	0.59

^a^ All results are adjusted for minimisation variables, and baseline utility for QALYs, M=20 multiple imputation sets, bootstrapped confidence intervals with 1000 replications, except results noted with ^b^

^b^ Unadjusted estimates with imputed data using M=20 multiple imputation sets

In THR, the INMB statistics are positive, even at the more stringent willingness-to-pay per QALY thresholds. The mean INMB for the NICE-recommended £20,000 per QALY threshold was of £1,125 (95 % BCI, £183 to £2,067). In TKR, our findings also indicate positive INMB statistics at all willingness-to-pay thresholds, but more uncertainty around these estimates, with all bootstrapped confidence intervals crossing the null.

**Fig. 1:**
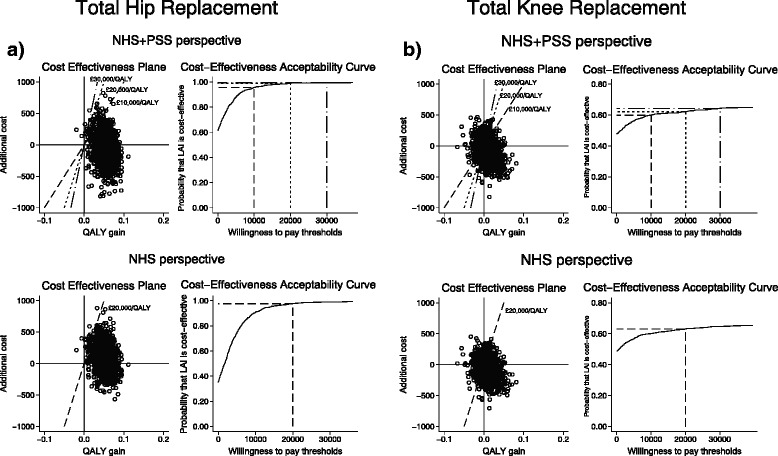
**a**. Total hip replacement: primary and secondary analyses cost-effectiveness plane and cost-acceptability curve. **b**. Total knee replacement: base care cost-effectiveness plane and cost-acceptability curve

Figure 1 plots the 1,000 replications of the adjusted bootstrapped incremental cost-effectiveness estimates in the cost-effectiveness planes and the corresponding CEACs for the various willingness-to-pay per QALY thresholds. Most estimates fall within the southeast quadrant of the plane, indicating the dominance of the intervention over standard care, more notably so for THR than TKR. The CEAC shows the uncertainty around the economic results, with a probability of LAI being cost-effective in TKR only slightly over 60 % at the £20,000 threshold. In THR, there is over 98 % probability of LAI being cost-effective at £20,000 per QALY and over 95 % at £10,000 per QALY.

### Economic results: NHS perspective

From an NHS perspective, LAI in addition to usual analgesia is no longer a dominant strategy in THR, albeit highly cost-effective, with an INMB of £961 (95 % BCI, £50 to £1,873; Table 6). There is considerably more uncertainty around the cost-effectiveness estimate in relation to decrease in pain severity measured by the WOMAC Pain scale at £16 per decrease in one point of pain (95 % CI, £–16,591 to £16,622). In TKR, LAI is still the cheapest and most effective intervention, in relation to both QALYs, with an INMB statistic of £260 (λ = 20,000; 95 % BCI, –£690 to £1,210, Table 7), and WOMAC pain, from an NHS perspective only.

### Sensitivity analysis results

**Fig. 2:**
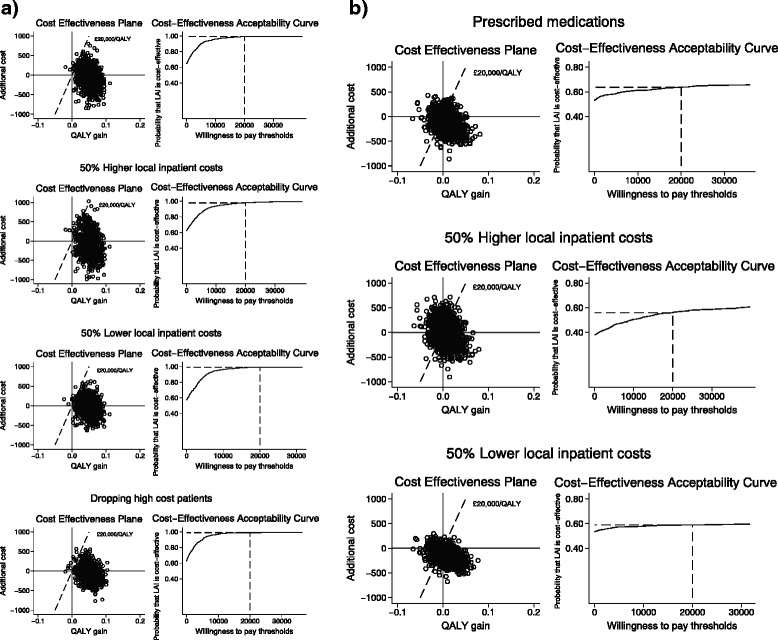
**a**. Total hip replacement: Sensitivity analyses cost-effectiveness planes and cost-acceptability curves. **b**. Total knee replacement: Sensitivity analyses cost-effectiveness planes and cost-acceptability curves

Our results in THR (Table 6) are robust to costing method of medication use, with an INMB statistic at the £20,000 per QALY threshold only slightly higher than base case. Varying local trust cost estimates during the initial patient stay by a factor of 50 % higher or lower did not alter our results, whereby the intervention is still dominant in both surgeries. In THR, the INMB statistics range from £1,051, using lower local costs, to £1,151, when higher local costs were used, compared with £1,125 in the base case. In TKR (Table 7), the respective figures are £253 and £159, compared with £264 in the base case. Due to changes in the components of the imputation model, QALY estimates vary slightly, particularly in TKR for these scenarios. In the scenario where we drop two high cost patients in THR, LAI is also the dominant treatment option with an INMB statistic of £1,121 (λ = 20,000; 95 % CI, £215 to £2,026). Figure 2a portrays the cost-effectiveness planes and CEACs for the scenarios, displaying probabilities of LAI being the cost-effective treatment option of over 98 % at the £20,000 per QALY threshold in THR. For TKR (Fig. 2b), sensitivity analysis results are consistent with base case results with just over 60 % probability of LAI being cost-effective at the £20,000 threshold.

## Discussion

Our findings suggest that administering LAI before wound closure is a cost-effective treatment option compared to current clinical analgesia regimens in both primary THR and TKR surgeries. The evidence is stronger for THR than TKR, with large positive INMB and a probability of LAI being cost-effective of over 95 % across all scenarios. There is no strong evidence for the positive INMB statistics for TKR, although results point to LAI being the dominant treatment option with a 62 % probability of being cost-effective at current NICE thresholds of £20,000 per QALY. There was little difference in costs between the two arms in both trials, with the cost-effectiveness results being driven by the QALY gain, which is larger in the THR trial. This higher QALY gain in the THR also accounts for the difference in economic results between trials. From an NHS perspective, in relation to THR, the intervention is no longer dominant, but still highly cost effective.

Our study is the first, to our knowledge, to report a trial based economic evaluation of LAI in the longer-term for patients receiving THR and TKR. The economic results of our trials reinforce the effectiveness results, and together provide evidence that the intervention is both effective and cost-effective in THR. There is more uncertainty around the effectiveness and cost-effectiveness results for TKR, where patients already benefit from a femoral nerve block in standard care. Given that there are no safety concerns with the treatment [27, 28], it should be recommended for use in patients having a THR.

Our study is not without limitations. The economic evaluation was carried out alongside the APEX trials, which were powered to detect a difference in the primary clinical outcome between treatment groups, but not in the cost-effectiveness outcomes. Collection of resource use data, particularly community-based health and social services, relied on patient-reported data, completed by postal questionnaires at three follow-up points. This led to a substantial amount of missing data and imputation was therefore needed. The imputed value estimates varied substantially from available case estimates. Patients with complete cost and QALY data had better pain outcomes. This was, however, accounted for in our imputation model, which included WOMAC Pain outcomes as a predictor of costs and QALYs, for a more conservative economic result. It would, therefore, be unlikely that the interventions would not to be cost-effective at current NICE recommended thresholds, even if complete cost and QALY data had been obtained. Local estimates for the initial hospital were used rather than national tariffs to allow for the disaggregation of this stay. This could potentially limit the generalisability of the results to other hospital locations. However, there were only minor differences in resource use in the micro-costed items. A sensitivity analysis which altered these local unit costs showed the initial results to be robust.

## Conclusions

Our findings suggest that administering LAI before wound closure is a cost-effective treatment option compared to current clinical analgesia regimens in both primary THR and TKR surgeries. The evidence, because of larger QALY gains, is stronger for THR. In TKR, there is more uncertainty around the economic result, and smaller QALY gains. Results, however, point to LAI being cheaper than standard analgesia, which already includes a femoral nerve block.

## Additional file

Additional file 1:
**Appendix 1.** CHEERS checklist.

## Abbreviations

APEX: The Arthroplasty pain experience (APEX) trials
CEAC: Cost-effectiveness acceptability curve
EQ-5D-3L: EuroQol questionnaire
ICER: Incremental Cost-effectiveness ratio
INMB: Incremental net monetary benefit
LAI: Local anaesthetic infiltration
PSS: Personal social service
QALY: Quality adjusted life year
THR: Total hip replacement
TKR: Total knee replacement
WOMAC: Western Ontario and McMaster Universities Osteoarthritis Index
